# Adding Metal Ions to the *Bacillus mojavensis* D50 Promotes Biofilm Formation and Improves Ability of Biocontrol

**DOI:** 10.3390/jof9050526

**Published:** 2023-04-28

**Authors:** Lining Zheng, Xuehu Gu, Liangpeng Sun, Meiqi Dong, Ao Gao, Zhe Han, Hongyu Pan, Hao Zhang

**Affiliations:** 1 College of Plant Protection, Jilin Agricultural University, Changchu 130118, China; 13074304872@163.com (L.Z.);; 2 College of Plant Sciences, Jilin University, Changchun 130062, China

**Keywords:** optimization, biofilm formation, *Bacillus mojavensis* D50, biocontrol effects, tomato gray mold

## Abstract

*Bacillus mojavensis* D50, a biocontrol strain, is used to prevent and treat the fungal plant pathogen *Botrytis cinerea*. *Bacillus mojavensis* D50’s biofilms can affect its colonization; thus, the effects of different metal ions and culture conditions on biofilm formation were determined in this study. The results of medium optimization showed that Ca^2+^ had the best ability to promote biofilm formation. The optimal medium composition for the formation of biofilms contained tryptone (10 g/L), CaCl_2_ (5.14 g/L), and yeast extract (5.0 g/L), and the optimal fermentation conditions included pH 7, a temperature of 31.4 °C, and a culture time of 51.8 h. We found that the antifungal activity and abilities to form biofilms and colonize roots were improved after optimization. In addition, the levels of expression of the genes *luxS*, *SinR*, *FlhA*, and *tasA* were up-regulated by 37.56-, 2.87-, 12.46-, and 6.22-fold, respectively. The soil enzymatic activities which related biocontrol-related enzymes were the highest when the soil was treated by strain D50 after optimization. In vivo biocontrol assays indicated that the biocontrol effect of strain D50 after optimization was improved.

## 1. Introduction

Tomato (*Solanum lycopersicon* L.), an important vegetable crop, is widely planted all over the world [1]. However, the occurrence of diseases can lead to a reduction in yields. Particularly important pathogens of tomato include the fungus *Botrytis cinerea*, which causes gray mold [2], and the bacterium *Ralstonia solanacearum*, which causes bacterial wilt [3]. Therefore, immense efforts and vast amounts of money are being invested to control these pathogens [4].

Currently, the primary way to control pathogens is the use of chemical pesticides [5]. They are inexpensive and highly efficient. However, the long-term use of chemical pesticides will lead to environmental pollution, pesticide resistance, and negative effects on human health [6,7]. Therefore, it is urgent to find an environmentally friendly approach to protect tomato plants from pathogens. Biological control is an approach to control plant diseases, which introduces another organism to control the pathogen rather than chemical pesticides. This approach is frequently highly effective, and it is used on many plants. The modes of action of biocontrol agents include space or nutrient competition, antibiotics production and inducible resistances. There are many studies about the use of biological control, such as a study which suggests that *Burkholderia cenocepacia* ETR-B22 could product volatile organic compounds and suppress the tomato gray mold [8]. *Bacillus velezensis* HY19 could also produce salicylic acid and numerous antifungal substances to inhibit the growth of tomato gray mold [9]. *Wickerhamomyces anomalus* could inhibit the growth of gray mold by competition for nutrients and space, and induce the host tissue’s disease resistance and producing of antifungal metabolites [10]. *Trichoderma harzianum* inhibited the tomato gray mold using competition for space [11].

Microbial biofilms are a microbial community that is attached to biological or abiotic surfaces. They can be composed of one or more microbial species [12]. Biofilm formation can improve the biocontrol effects on pathogens. The formation of biofilm can help the bacteria to rapidly ingest nutrition and become more competitive. In addition, it can also facilitate colonization of the plant surface, which can protect the plant from pathogens [13]. Many studies have reported that the amount of metal ions and culture conditions can influence the formation of biofilm. Yang et al. [14] reported that the morphology of the *B. subtilis* 1JN2 biofilm was flattened and the expression of biofilm related genes was down-regulated under high concentrations of Cd^2+^, while the addition of Mg^2+^ increased the biocontrol effect of *Burkholderia pyrrocinia* JK-SH007 [15]. The three *Bacillus* spp. strains which could form biofilm were isolated to protect maize from pathogens, the temperature, culture medium, and culture time, which could influence the biofilm formation and thus further impact the biocontrol effect of these strains [16]. The application of phenylalanine could promote the biofilm formation of *Meyerozyma caribbica* and improved its biocontrol efficacy [17]. These studies have indicated that the biofilm formation of strains can influence their biocontrol effects.

The *luxS*/AI-2 quorum sensing (QS) system is an important factor affecting biofilm formation [18]. Many studies have reported that the gene of *luxS* influences the formation of biofilms, such as *Shewanella xiamenensis* [19] and *Lactobacillus reuteri* [20]. In addition, the QS system can also regulate the probiotic activities of lactic acid bacteria [21]. The transcriptional repressor *sinR* is a master regulator of biofilm formation and the production of the secreted, amyloid-like protein component of the matrix (*TasA*), thus blocking biofilm formation [22]. The gene of *flhA*, encoded flagellin protein *FlhA*, can influence the formation of biofilm. Minamino et al. [23] have reported that the gene of *flhA* can change the growth of flagella, further influencing biofilm formation. The *tasA* is a major gene which encodes the protein involved in antimicrobial activities, spore coat assembly, and germination. It is also found in the stationary phase, sporulating cultures, and the biofilm matrix [24].

*B. mojavensis* D50, which was isolated from tomato rhizosphere soil, has been reported to have a substantial effect on *B. cinerea*. Moreover, its fermentation supernatant also had great antifungal effect on tomato gray mold [5]. However, there have been few studies on the promotion of biofilm formation and biocontrol effects by the manipulation of concentrations of metal ions and culture conditions. In this study, to further determine the effect of biofilm formation on pathogen inhibition, the goals were as follows: (1) to screen the optimal concentration of metal ions and culture conditions for biofilm formation; (2) to determine the effect of optimal metal ion and culture conditions on the levels of expression of the genes related to biofilm formation; (3) to assess the effect of optimal metal ion and culture conditions of *B. mojavensis* D50 on soil enzyme activities; and (4) to assess the effect of optimal metal ion and culture conditions of *B. mojavensis* D50 on reducing tomato grey mold.

## 2. Materials and Methods

### 2.1. Strains, Medium, Culture Conditions and Plant Materials

*Bacillus mojavensis* D50, isolated from tomato rhizosphere soil, was used to prevent tomato from *Botrytis cinerea. B. mojavensis* D50 and *B. cinerea* were kindly provided by the Laboratory of Pesticide Bioassay, Plant Protection College at Jilin Agricultural University (Jilin, China). The LB broth that was used to culture *B. mojavensis* D50 contained 5 g yeast powder, 10 g tryptone, 10 g NaCl, and 1000 mL distilled water. The potato dextrose agar (PDA) medium that was used to culture *B. cinerea* contained 20 g glucose, 20 g agar, 200 g potato, and 1000 mL distilled water.

The initial culture conditions that were used to optimize the formation of biofilm were 30 °C, a culture time of 48 h, and pH of 7. The initial medium that was used to optimize the formation of biofilm formation was LB broth (excluding metal ions) to exclude the influence of metal ions on biofilm formation: 5 g/L yeast powder, 10 g/L tryptone, and 1000 mL water. The tomato plants were planted as described by Zheng et al. [25], the seeds were sterilized in 0.5% sodium hypochlorite solution for 1 min and rinsed with sterile distilled water three times, then the seeds were incubated at 28 °C for 4 days for germination. One seedling was planted in each plastic pot, and the tomato plants were grown in a greenhouse at 25–30 °C with a 14 h-light/10 h-dark cycle and 70% relative humidity with regular irrigation.

### 2.2. Preparation of the B. mojavensis D50 Suspension

To prepare the *B. mojavensis* D50 suspension, a single colony was inoculated into a 250-mL Erlenmeyer flask that contained 100 mL of LB broth, and it was shaken at 150 rpm and cultivated at 30 °C for 12 h. The suspension was centrifuged at 7000 rpm for 10 min, and the strains were washed three times with PBS (pH 7.3). The suspension was adjusted to 1.0 × 10^8^ cfu/mL [25].

### 2.3. Semiquantitative Evaluation of the B. mojavensis D50 Biofilm

The crystal violet method was used to determine the amount of biofilm formed [26]. Briefly, a suspension of *B. mojavensis* D50 was prepared as described in Section 2.2. A volume of 5 μL/well was added to the wells in a 96-well plate and mixed with the relevant medium (95 μL/well). The plate was left stationary for cultivation. After that, the cell culture was removed and dyed with 1% crystal violet (CV, 100 μL/well) for 15 min. The plate was washed three times with distilled water to remove excess dye. The biofilm was extracted with absolute ethanol (200 μL/well), and the amount of biofilm formation was determined at 570 nm [27].

### 2.4. Screening of the Optimal Metal Ions and Conditions for Biofilm Formation

#### 2.4.1. Determination of the Effect of Metal Ions on Biofilm Formation by a Single Factor Experiment

To determine the effect of metal ions on biofilm formation, the “one-factor-at-a-time” method was also used in this experiment. Briefly, the CaCl_2_, MnCl_2_, FeCl_3_·6H_2_O, MgSO_4_·7H_2_O and NaCl were added into initial medium. The concentrations of CaCl_2_, MnCl_2_, FeCl_3_·6H_2_O, MgSO_4_·7H_2_O were adjusted to 0 g/L, 5 g/L, 10 g/L, 15 g/L, 20 g/L, and 25 g/L, respectively, and the concentrations of NaCl were adjusted to 0 g/L 20 g/L, 25 g/L, 30 g/L, 35 g/L, and 40 g/L, respectively. The *B. mojavensis* D50 suspension (5 μL) was inoculated into the medium (which contained different metal ions) and cultured at 30 °C for 48 h. The amount of biofilm formation was determined as described in Section 2.3 [15]. The experiments were replicated three times and repeated three times.

#### 2.4.2. Determination of the Effect of Culture Conditions on Biofilm Formation by a Single Factor Experiment

The “one-factor-at-a-time” method was also used in this experiment. Briefly, the culture conditions were established as follows: the pH of initial medium was adjusted to 5, 6, 7, 8, 9, and 10. The culture time was adjusted to 12 h, 24 h, 36 h, 48 h, 60 h, and 72 h. The culture temperature was incubated to 25 °C, 30 °C, 35 °C, 40 °C, and 45 °C. The amount of biofilm formed was determined as described in Section 2.3. The experiments were replicated three times and repeated three times.

#### 2.4.3. Determination of the Optimal Amount of Metal Ions and Culture Conditions for Biofilm Formation Using a Box-Behnken Design

The results of the single factor experiment indicated the three factors that had the best effect on biofilm formation, and they were selected for coding. OD_570_ was used as the response value. A Box-Behnken central combined experiment was used to design the response surface test. The codes and levels of each factor are shown in Table 1. The Box-Behnken design (BBD) was used to conduct 17 tests, including five tests to estimate the error (Table 2). The concentration of metal ions and culture conditions were prepared based on the BBD. The ability to form biofilm was measured as described in Section 2.3. Finally, the prediction of metal ions and culture conditions were verified under the same fermentation conditions as the BBD. Based on the response surface methodology (RSM) results, the final metal ions and culture conditions were determined and verified experimentally [28,29]. The experiments were replicated three times and repeated three times.

### 2.5. Effect of Metal Ions and Culture Conditions on the Biocontrol Characteristics of B. mojavensis D50 against B. cinerea In Vitro

To determine the effect of metal ions and culture conditions on the characteristics of biocontrol, the flat-stand method was used to measure the antifungal activity in this experiment [30]. As described by Fu et al. [15], the ability to form biofilm and root colonization by *B. mojavensis* D50 before and after optimization were also measured. The experiments were replicated three times and repeated three times.

### 2.6. B. mojavensis D50 Biofilm-Related Analysis of Differentially Expressed Genes

To determine the effect of optimized metal ions and culture conditions on the differential expression of genes related to biofilm formation, the *B. mojavensis* D50 suspension was inoculated into a 250-mL Erlenmeyer flask that contained 100 mL of optimized culture medium. The culture was then cultivated at 31.4 °C and 150 rpm for 12 h and 51.8 h. The key genes related to biofilm formation, *luxS*, *SinR*, *FlhA*, and *tasA*, were analyzed. The primers were designed using Primer 6 software (Table S1), and the total RNA was extracted using a Trazol up Plus RNA Kit (TransGen Biotech., Beijing, China). The cDNA was generated according to the manufacturer’s instructions for the TransGen all-in-one first-strand cDNA synthesis SuperMix (TransGen Biotech.). The *luxS*, *SinR*, *FlhA*, and *tasA* genes were measured in this study. Quantitative real-time PCR (qRT-PCR) was performed using PerfectStart Green qPCR SuperMix on a Roche Light Cycler^®^ 96 (Roche Diagnostics Corporation, Indianapolis, IN, USA). The qRT-PCR program used was as follows: 95 °C for 2 min, followed by 32 cycles of 95 °C for 30 s, 60 °C for 30 s, 72 °C for 30 s and 78 °C for 11 s. The melting curve cycle program was as follows: 95 °C for 10 s, 60 °C for 1 min, and 97 °C for 1 s. The relative levels of expression of the genes were determined using the 2^−ΔΔCT^ method, and the *16S rDNA* gene was used as a reference gene. Data are reported on a logarithmic scale as a relative gene expression ratio (RQ) after calibration on values obtained at *B. mojavensis* D50 that was cultured in basic medium [31]. The experiments were replicated three times and repeated three times.

### 2.7. Effect of Metal Ions and Culture Conditions on the Biocontrol Characteristics of B. mojavensis D50 against B. cinerea In Vivo

As described by Zheng et al., the tomato plant and spore suspension of *B. cinerea* were prepared [25]. The suspension of *B. mojavensis* D50 before or after optimization was prepared as described in Section 2.2. The 6-leaf-stages of tomato seedlings were used in this experiment. The suspension of *B. mojavensis* D50 before or after optimization (1.0 × 10^8^ cfu/mL, 15 mL/pot) was irrigated into the roots of tomato plants as the treatment, whereas the sterile water (15 mL/pot) was irrigated into the roots as the control. After 7 days, the spores (5 *×* 10^4^ spores/mL, 8 mL/pot) were sprayed on the tomato seedlings. The tomato seedlings were incubated in a growth chamber at 26 ± 0.5 °C and covered with plastic bags for 3 days to maintain high humidity. The disease incidence was determined 7 days after inoculation with *B. cinerea* [32,33]. Each treatment contained 16 pots with 1 seedling per pot. The experiments were replicated three times and repeated three times. The biochemical growth parameters of tomato plant were measured as described by Zhang et al. [31].

### 2.8. Effect of B. mojavensis D50 on Soil Enzyme Activities before and after Optimization

To determine the effect of optimized metal ions and culture conditions of *B. mojavensis* D50 on soil enzyme activities, the activities of invertase, catalase, urease, and dehydrogenase were measured. The treatments were the same as those described in Section 2.7. The soil samples were collected at 2 h and 1, 3, 5, 7, and 15 days. Invertase activity was measured by the 3,5-dinitrosalicylic acid method. Briefly, 2 g of soil sample was mixed with 15 mL of 8% sucrose solution, 5 mL of PBS (pH 5.5), and 0.2 mL drops of toluene, and it was cultured at 37 °C for 24 h. A volume of 1 mL of the supernatant was mixed with 5 mL of 3,5-dinitrosalicylic acid and 5 mL of water, and the invertase activity was determined at 508 nm [34]. To measure the catalase activity, 2 g of soil sample was mixed with 40 mL water and 5 mL of 0.5% H_2_O_2_ and incubated at 30 °C for 20 min. A volume of 5 mL of H_2_SO_4_ (1.5 mol/L) was added to terminate the reaction. Finally, the supernatant was titrated with KMnO_4_ (20 mmol/L) [35]. Urease activity was determined by the Berthelot method. Briefly, 5 g of soil was mixed with 1 mL of PBS (pH 7.7, 20 mmol/L) and then incubated at 37 °C for 2 h. It was extracted with KCl (2 mmol/L), and the absorbance at 690 nm was measured using a visible spectrophotometer [36]. The dehydrogenase activity was determined by colorimetry. Briefly, 2 g of soil was mixed with 2 mL of tetrazolium chloride (TTC) and 2 mL water and then cultured at 37 °C for 24 h. The solution was extracted with 5 mL of alcohol. Its absorbance was measured at 485 nm using a visible spectrophotometer. The experiments were replicated three times and repeated three times.

### 2.9. Statistical Analysis

The data were subjected to analyses of variance (ANOVA) using SPSS 26.0 (IBM, Inc., Armonk, NY, USA). The means were separated by a least significant difference (LSD) test at the level *p* < 0.05. Respective significant differences were denoted using different letters (a, b, c, etc.).

## 3. Results

### 3.1. Screening of the Optimal Metal Ions and Culture Conditions for Biofilm Formation

**Figure 1:**
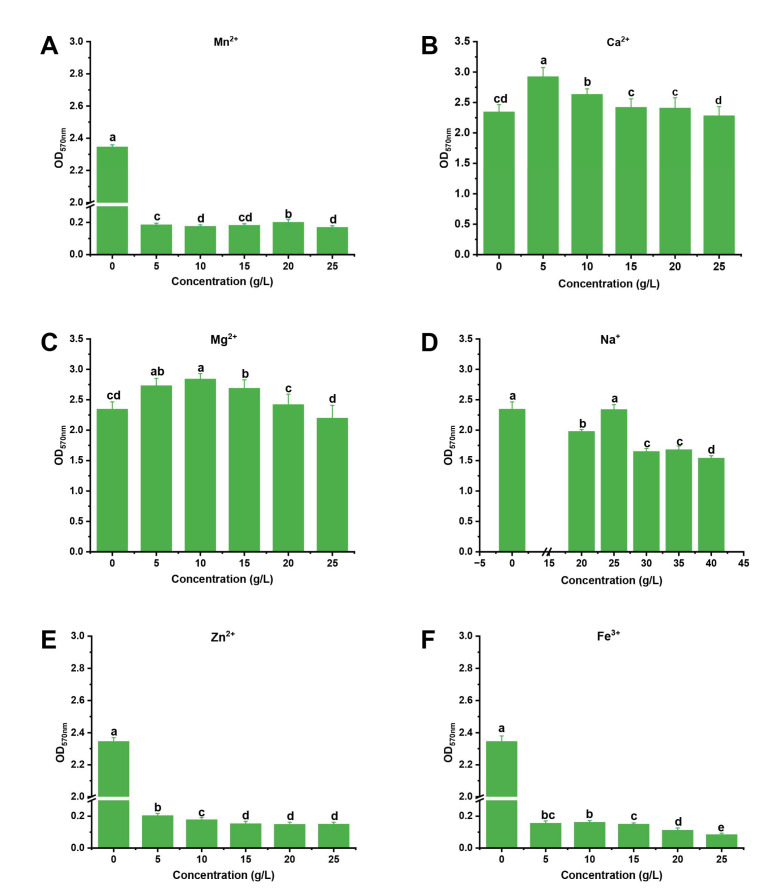
Effect of different concentrations of metal ions on *B. mojavensis* D50 biofilm formation: Mn^2+^ (**A**), Ca^2+^ (**B**), Mg^2+^ (**C**), Na^+^ (**D**), Zn^2+^ (**E**), and Fe^3+^ (**F**). The strain D50 (5 μL) was inoculated into mediums (containing different metal ions) and cultured at 30 °C for 48 h. Bars followed by the same letter are significantly different at *p* < 0.05 using a least significant difference (LSD) test. Error bars indicate the SD of three experiments. SD, standard deviation.

**Figure 2:**
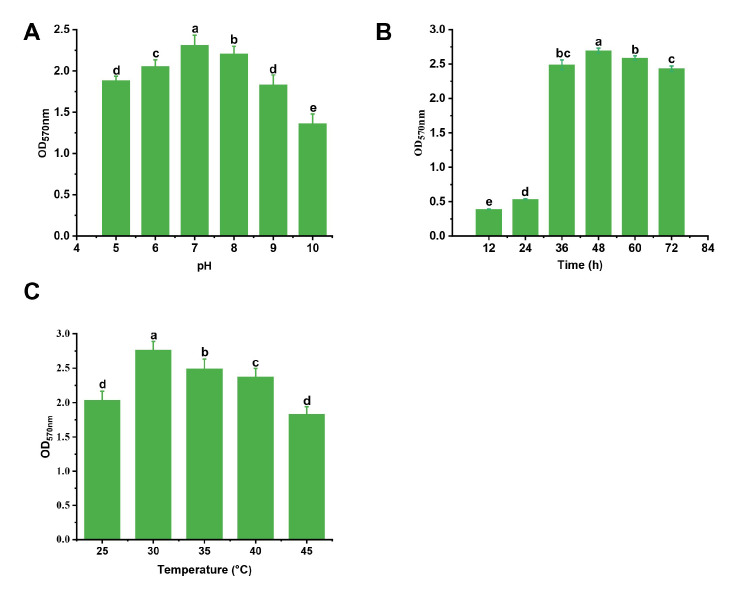
Effect of different concentrations of culture conditions on *B. mojavensis* D50 biofilm formation: pH (**A**), time (**B**), and temperature (**C**). The strain D50 (5 μL) was inoculated into mediums (without metal ions) and cultured for different amounts of time and at different temperatures. To determine the effect of pH on biofilm formation of strain D50, the strain D50 (5 μL) was inoculated into mediums (of different pH) and cultured at 30 °C for 48 h. Bars followed by the same letter are significantly different at *p* < 0.05 using a least significant difference (LSD) test. Error bars indicate the SD of three experiments. SD, standard deviation.

The “one-by-one factor” experiments were used to select the metal ions and conditions. We found that the Ca^2+^ and Mg^2+^ could promote the formation of biofilm and the Na^+^ had less of an effect on the biofilm formation, while Mn^2+^, Fe^3+^, and Zn^2+^ inhibited it (Figure 1). When the concentrations of Ca^2+^ and Mg^2+^ were 5 g/L and 10 g/L, respectively, the biofilm formation was maximum: the biofilm formation increased by 24.7% and 21.1%, respectively. We found that the factors that were the most effective at stimulating biofilm formation were CaCl_2_ (5 g/L), pH 7, and a culture time of 48 h at 30 °C (Figure 1 and Figure 2). According to the principle of Box-Behnken design, the optimal concentration or conditions were taken to the climbing test as the central point of the response surface test factor level. A response surface analysis (three factors and three levels) was used to determine the optimal level of the primary factors that affected the biofilm formation of *B. mojavensis* D50. The design factors and levels of the Box-Behnken response surface are shown in Table 1, and the design results are shown in Table 2. A regression analysis of the results in Table 3 was used to obtain the regression equation of Y = 3.19 + 0.0129A + 0.0253B + 0.0456C − 0.0213AB + 0.0075AC + 0.0297BC − 0.2229A^2^ − 0.2882B^2^ − 0.2984C^2^. The correlation coefficient of the model was R^2^ = 0.9710, and the corrected determination coefficient was R^2^_Adj_ = 0.9336. The fitting model could clarify the change of 93.36% of the response value, indicating that there was a strong fitting degree between the experimental data and that predicted. There was little experimental error, which indicated that it could be used to predict and analyze the factors that affected the biofilm formation.

**Figure 3:**
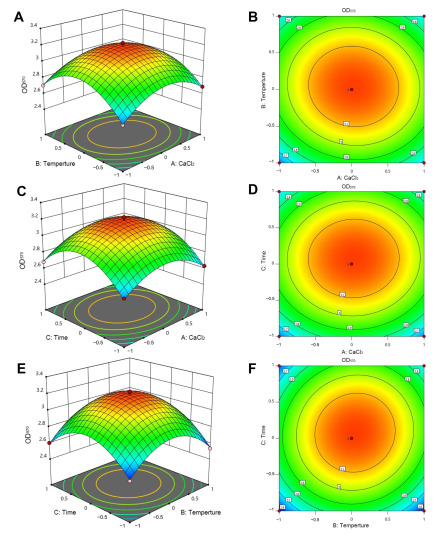
Three-dimensional response and two-dimensional surface contour for biofilm formation amount as evaluation indicators. The interactions between solution concentration of CaCl_2_ and temperature (**A**,**B**); solution concentration of CaCl_2_ and time (**C**,**D**); and temperature and time (**E**,**F**). The change of color from blue to red in the graph indicates a change in extraction quality from less to more, and the faster the change, the greater the slope, which has a more significant impact on the experimental results.

**Figure 4:**
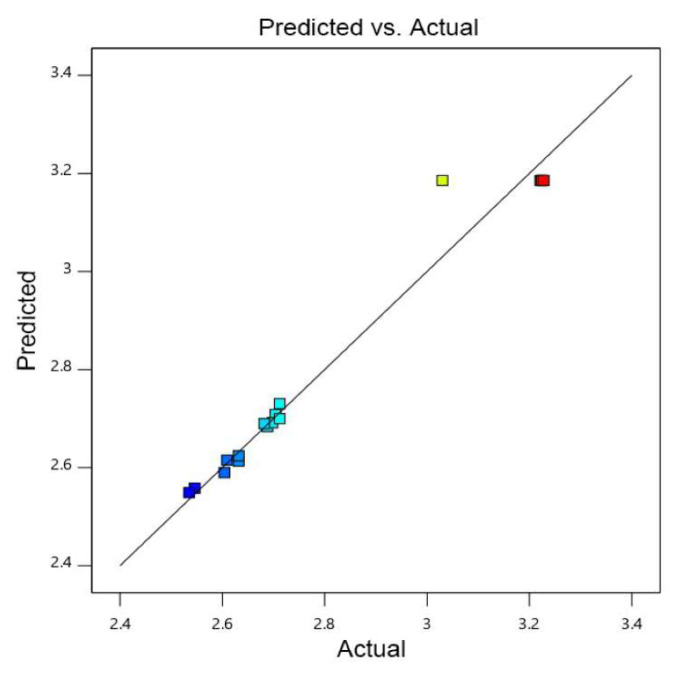
Parity plot of correlation between the actual value and predicted value of biofilm formation. The change of color from blue to red in the graph indicates a change in extraction quality from less to more, and the faster the change, the greater the slope, which has a more significant impact on the experimental results.

The variance analysis of response surface quadratic model is shown in Table 3. The response surface quadratic model was highly significant (*p* < 0.0001), and the lake of fit (lake of fit = 0.0710 > 0.05) was not significant. There were no abnormal points in the data, and there was a high fitting degree of the regression equation. The influence degree of each factor of *F* value on the response value, C > B > A, indicated that the influence intensity of each factor on the biofilm formation was as follows: time > temperature > CaCl_2_. The response surface curve and contour map were drawn based on the results of the equation simulation (Figure 3). All three surface graphs were convex, and all the contour lines were oval. These results indicated that there was a stable maximum value of the response. As shown in Figure 3 and Table 3, the simulation factors A^2^, B^2^, and C^2^ were significant, indicating that magnesium sulfate had a significant impact on the biofilm formation. In contrast, A, B, and C were not significant, indicating that CaCl_2_, temperature, and time had no significant impact on the biofilm formation, and the impact of various influencing factors on biofilm formation was not linear. AB, AC, and BC were not significant, indicating that the interaction between various factors was not significant. The parity plot of biofilm formation shows the distribution of predicted yield and actual yield of biofilm formation under different conditions, as seen in Figure 4. The corresponding predicted data of the model was almost consistent with that of the actual data, indicating that the polynomial model was highly accurate and universal. Thus, it was reasonable to use the model to analyze the corresponding trend.

### 3.2. Determination of Antagonistic Abilities and Colonization Capacity Differences

**Figure 5:**
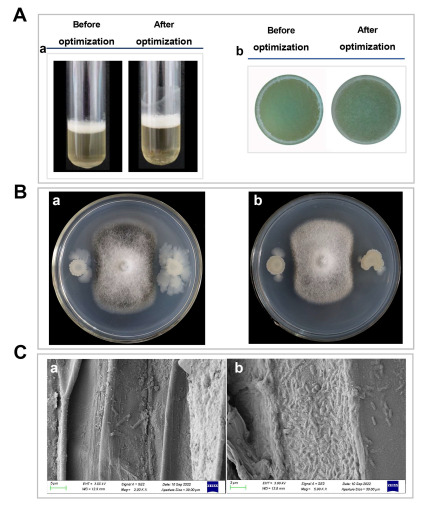
Effect of *B. mojavensis* D50 on biofilm formation, antifungal activity, and colonization ability on the plant root. (**A**) The biofilm formation of D50 ((a): before optimization, and (b): after optimization). The strain D50 (5 μL) was inoculated into initial or optimum medium and cultured at 30 °C or 31.4 °C for 48 h or 51.8 h. (**B**) The antifungal activity of D50 ((a): before optimization, (b): after optimization). The strain D50 (5 μL) which was cultured into initial or optimum medium was inoculated into PDA medium and cultured at 26 °C for 7 days to determine the biocontrol effect. (**C**) The colonization ability of D50 ((a): before optimization, (b): after optimization). The strain D50 (1.0 *×* 10^8^ cfu/mL, 20 mL), before or after optimization, was poured on the roots of tomatoes, and the colonization ability was determined after 2 days.

Antagonistic abilities and colonization capacity are important targets to optimize for high biocontrol ability. Therefore, these abilities were measured. As shown in Figure 5A, the biofilm of *B. mojavensis* D50 (after optimization) was more viscous and denser than those in *B. mojavensis* D50 (before optimization). The biofilm yields of *B. mojavensis* D50 (after optimization) were more than those in *B. mojavensis* D50 (before optimization). The antagonistic abilities were improved after optimization, and the morphology of *B. cinerea* obviously changed (Figure 5B). After optimization, the colonization capacity of *B. mojavensis* D50 increased in the root of the tomato plant (Figure 5C). These results could indicate that the biocontrol ability of *B. mojavensis* D50 increased after optimization.

### 3.3. B. mojavensis D50 Biofilm-Related Gene Differential Expression Analysis

**Figure 6:**
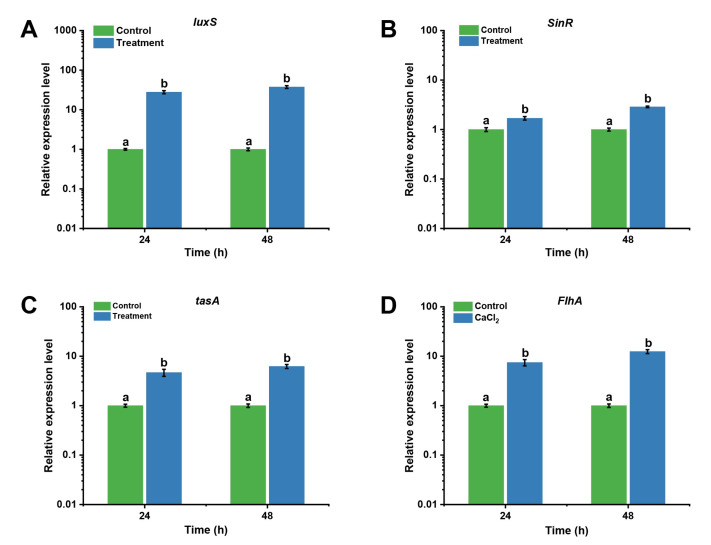
The levels of expression of the genes *luxS*, *SinR*, *FlhA*, and *tasA* related to biofilm formation were detected using qRT-PCR: (**A**) *luxS*, (**B**) *SinR*, (**C**) *tasA*, (**D**) *FlhA*. *B. mojavensis* D50 (1.0 *×* 10^8^ cfu/mL, 1 mL) was inoculated into initial medium (100 mL) and cultured at 30 °C for 48 h as the control, while *B. mojavensis* D50 (1.0 *×* 10^8^ cfu/mL, 1 mL) was inoculated into optimization medium (100 mL) and cultured at 31.4 °C for 51.8 h as the treatment. Bars followed by the same letter are significantly different at *p* < 0.05 using a least significant difference (LSD) test. Error bars indicate SD of three experiments. qRT-PCR, quantitative PCR; SD, standard deviation.

To determine the differential expression of biofilm-related genes (before or after optimization), the levels of expression of the genes *luxS*, *SinR*, *FlhA*, and *tasA* were measured. As shown in Figure 6, the level of expression of the *luxS* gene was upregulated by 27.87- and 37.56-fold when the culture times were 24 h and 51.8 h, respectively (Figure 6A). The level of expression of the *SinR* gene after optimization was higher than that before optimization at the different culture times (1.69- and 2.87-fold) (Figure 6B). Moreover, the level of expression of the *FlhA* gene was upregulated by 7.47- and 12.46-fold when the culture times were 24 h and 51.8 h, respectively (Figure 6C). The level of expression of the *tasA* gene after optimization was higher than that before optimization at the different culture times (4.68- and 6.22-fold, respectively) (Figure 6D). These results indicated that the levels of expression of biofilm-related genes were upregulated after optimization, and the ability to form biofilm improved owing to the increased expression of the genes involved in the production of biofilm.

### 3.4. Effect of Metal Ions and Culture Conditions on the Biocontrol Characteristics of B. mojavensis D50 against B. cinerea In Vivo

Furthermore, *B. mojavensis* D50 (before and after optimization) was used in this experiment to assess its antifungal ability improvement. Three days after inoculation with *B. cinerea*, small spotted lesions appeared on the tomato. As time went on, lesions became larger and caused extensive yellowing of leaves in the group BS. In the group BSA and BSB, small spotted lesions appeared after five days’ inoculation with *B. cinerea*. The lesions did not expand with time. After the inoculation with *B. cinerea* for seven days, large areas of tomato plants in the group BS turned yellow, while a small portion of the plant had diseased spots in the group BSB and BSA. The disease severity index was 85.6 in the group BS (*B. cinerea* + sterile water). In the group BSB (*B. cinerea* + strain D50 before optimization), the severity of disease and disease reduction was 58.4 and 39.7, respectively, while in the group BSA (*B. cinerea* + strain D50 after optimization), the severity of disease and disease reduction was 45.3 and 46.2, respectively (Table 4).

As shown in Table 5, various growth and biochemical parameters of the tomato seedlings were measured. The fresh weight, shoot length, and chlorophyll content in the tomato seedlings differed significantly among BS (*B. cinerea* + sterile water), BSB (*B. cinerea* + strain D50 before optimization), and BSA (*B. cinerea* + strain D50 after optimization) (*p* < 0.05). The chlorophyll content, shoot length, and fresh weight in BSA were higher than those in BS and BSB. Moreover, the contents of chlorophyll a, chlorophyll b, and total chlorophyll in BSA were significantly higher than BS (24.2%, 22.4%, and 23.8%, respectively) and BSB (19.3%, 9.8%, and 12.8%, respectively). The total phenolic content was enhanced by 52.5% and 10.3% more than BS and BSB, respectively. The correlated parameters (chlorophyll a, chlorophyll b, total chlorophyll, fresh weight, shoot length, root length, total phenolic content, and total soluble protein) in BS and BSB were lower than that in BSA. This effect was associated with decreases in fresh weight, shoot length, and root length. The results indicated that the biofilm formation improvement could improve the antifungal effects in vivo.

### 3.5. Effect of B. mojavensis D50 (before and after Optimization) on Soil Enzyme Activities

**Figure 7:**
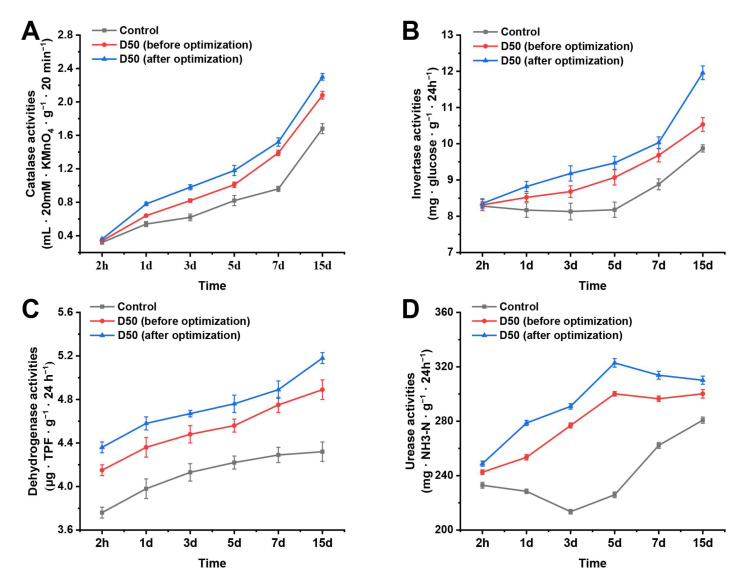
Soil enzyme activities in various treatments (sterile water as control; *B. mojavensis* D50 before or after optimization as treatment): (**A**) catalase, (**B**) invertase, (**C**) dehydrogenase, and (**D**) urease. The soil was collected at 2 h, 1 day, 3 days, 5 days, 7 days, and 15 days after the inoculation with strain D50 (before and after optimization). Error bars indicate SD of three experiments. SD, standard deviation.

To determine the effect of *B. mojavensis* D50 (before or after optimization) on soil enzyme activities (related to biocontrol), the activities of invertase, catalase, urease, and dehydrogenase were measured. As shown in Figure 7, the invertase, catalase, and dehydrogenase activities increased from 2 h to 15 days (Figure 7A,C,D). The urease activity in BSB (*B. cinerea* + strain D50 before optimization) and BSA (*B. cinerea* + strain D50 after optimization) group increased from 2 h to 5 days, while it decreased from 5 days to 15 days. In addition, the urease activity in the BS (*B. cinerea* + sterile water) group decreased from 2 h to 3 days, while it decreased from 3 days to 15 days (Figure 7B). The soil enzyme activities in the T3 group were the highest among these groups (Figure 7) (*p* < 0.05). These results indicated that *B. mojavensis* D50 (after optimization) improved the related biocontrol soil enzyme activities and promoted the growth of tomato plants.

## 4. Discussion

Most Gram-positive bacteria can form biofilms, but this process is difficult to observe under culture conditions [15]. The formation of biofilm facilitates its colonization in plants and increases its biocontrol ability [37]. In this study, the metal ion and culture conditions were optimized by the one-by-one method and response surface methodology. As shown in Figure 1 and Figure 2, Ca^2+^ promoted biofilm formation, and the temperature and culture time were also important factors that influenced the biofilm formation. The optimized culture conditions and culture medium were determined using response surface methodology. Many studies have reported that the optimal culture medium and conditions can influence biofilm formation; for instance, the biofilm formation of *B. subtilis* 1JN2 decreased when it was cultured under a high concentration of Cd^2+^ [14]. The culture medium and culture time could also impact the biofilm formation of *Bacillus* spp. [16]. Glycerol, as a carbon source, and Ca^2+^ and Mg^2+^ have been reported to have a substantial effect on biofilm formation [15]. These studies indicated that optimizing the culture conditions and culture medium can improve the biofilm formation, and their results were consistent with ours.

Biofilm is an aggregated form of a growing microorganism. Biofilm formation can influence strain colonization, affecting its biocontrol effects [37]. To ensure the effect of optimizing the culture medium and culture conditions on biofilm formation, the antifungal abilities and colonization capacity were measured. We found that these biocontrol characteristics were improved (Figure 5). Many studies have reported that the biocontrol effects could improve by enhancing the biofilm formation [38]. Our results closely agreed with the findings in these studies.

The quorum sensing (QS) system, which regulates biofilm formation, is one of the important factors that regulates biofilm formation in many bacteria [39]. To determine the mechanism of biofilm formation, the levels of expression of the genes *luxS*, *SinR*, *FlhA*, and *tasA* involved in its formation were measured. We found that the level of expression of these genes increased after optimization. These results showed that the optimal culture medium and conditions could upregulate the level of expression of the biofilm-related genes to promote biofilm formation (Figure 6). The optimal culture medium and conditions of *C. albicans* could promote biofilm formation and growth [40]. These results were consistent with ours.

To determine the effect of *B. mojavensis* D50 in vivo, the disease incidence and severity, fresh weight, shoot length, and chlorophyll content of the tomato seedlings were measured. We found that *B. mojavensis* D50 (after optimization) effectively protected the tomato plants from *B. cinerea*. In addition, the growth and biochemical parameters of the tomato seedlings treated with *B. mojavensis* D50 (after optimization) were the highest among those in BS and BSB group (*p* < 0.05) (Table 5). There are some studies on the effect of biocontrol strain on pathogens, such as the ability of *B. subtilis* Y2 to inhibit the growth of *Alternaria brassicicola* in pear fruit [41]. The antagonistic activity of *B. velezensis* CMRP 4489 could inhibit the growth of pathogens by the formation of biofilm [42].

Soil enzyme activity is an important physical and chemical index of soil. It is generally believed that a higher activity of soil enzymes in the soil environment indicates that the soil is more fertile [43]. The results of this study showed that the activities of urease, invertase, catalase, and dehydrogenase in the soil treated with *B. mojavensis* D50 (after optimization) increased in the tomato rhizosphere (Figure 7). Simultaneously, they could promote the growth of root length, root weight, and fresh weight of tomato seedlings, indicating that the growth promotion of *B. mojavensis* D50 could be related to its ability to improve the activities of soil enzymes, which improves the fertility of soil.

## 5. Conclusions

In this study, the effects of different metal ions and culture conditions on biofilm formation were determined. After optimization, the characteristics of biocontrol and the ability of colonization were improved, and the levels of expression of the biofilm-related genes *luxS*, *SinR*, *FlhA*, and *tasA* were up-regulated. After optimization, *B. mojavensis* D50 could increase the activity of soil enzymes related to biological control, and the biocontrol effect was also improved in vivo. Further study is needed with a focus on searching for how the metal ions influence the biofilm formation of *B. mojavensis* D50, and the effect of key genes which are related to the biofilm formation and biocontrol of *B. cinerea*.

## Figures and Tables

**Table 1:** Box-Behnken design to optimize the biofilm formation medium and conditions using three components at three levels.

Design Variable (Factors)	Unit	Code	Real Values of the Coded Levels		
			−1	0	+1
CaCl_2_	g/L	A	0	5	10
Temperature	℃	B	25	30	35
Time	h	C	36	48	60

Note: “+1” represents high level, “−1” represents low level.

**Table 2:** Box Behnken design scheme containing 17 experimental runs.

Runs	A	B	C	OD_570_
1	0	−1	−1	2.546
2	0	0	0	3.224
3	0	1	−1	2.535
4	−1	0	−1	2.632
5	0	−1	1	2.604
6	0	0	0	3.226
7	−1	−1	0	2.609
8	−1	1	0	2.704
9	0	0	0	3.228
10	1	1	0	2.698
11	0	1	1	2.712
12	1	0	1	2.712
13	1	−1	0	2.688
14	0	0	0	3.221
15	0	0	0	3.03
16	−1	0	1	2.682
17	1	0	−1	2.632

Note: “+1” represents high level, “−1” represents low level.

**Table 3:** ANOVA for response surface reduced quadratic model.

Source	Sum of Squares	df	Mean Square	*F*-Value	*p*-Value	
Model	1.07	9	0.1189	26.01	0.0001	Significant
A-CaCl_2_	0.0013	1	0.0013	0.2902	0.6068	
B-Temperature	0.0051	1	0.0051	1.12	0.03258	
C-Time	0.0167	1	0.0167	3.64	0.0979	
AB	0.0018	1	0.0018	0.3953	0.5495	
AC	0.0002	1	0.0002	0.0492	0.8307	
BC	0.0035	1	0.0035	0.7748	0.4079	
A^2^	0.2092	1	0.2092	45.78	0.0003	
B^2^	0.3496	1	0.3496	76.51	<0.0001	
C^2^	0.3749	1	0.3749	82.05	<0.0001	
Residual	0.0320	7	0.0046			
Lack of Fit	0.0016	3	0.0005	0.0710	0.9724	Not significant
Pure Error	0.0304	4	0.0076			
Cor Total	1.10	16				

Note: ANOVA, analysis of variance.

**Table 4:** Antifungal spectra of *B. mojavensis* D50 (before and after optimization) against *Botrytis cinerea* in vivo. Sterile water as control; *B. mojavensis* D50 before or after optimization as treatment.

Treatment	Inoculants of Treatment	Disease Severity Index (%)	Disease Reduction (%)
BS	*B. cinerea* + Sterile water	85.6	-
BSB	*B. cinerea* + strain D50 (before optimization)	58.4	39.7
BSA	*B. cinerea* + strain D50 (after optimization)	45.3	46.2

Data with sample size n = 16 plants per treatment.

**Table 5:** Growth and biochemical parameters (fresh weight, root length, shoot length, chlorophyll a, chlo-rophyll b, total chlorophyll, total phenolic content, and total soluble protein) of tomato plants after strain D50 (before and after optimization) treatments. Bars followed by the same letter are significantly different at *p* < 0.05 by LSD test, error bars indicate ± SD of triplicates.

Treatments	Fresh Weight (g)	Root Length (cm)	Shoot Length (cm)	Chl a (mg/mL)	Chl b (mg/mL)	Total Chlorophyll (mg/mL)	Total Phenolic Content (mg/100 g)	Total Soluble Protein (mg/100 g)
BS	1.45 ± 0.05 c	6.15 ± 0.13 c	17.82 ± 0.32 c	11.99 ± 0.08 c	3.75 ± 0.08 c	15.74 ± 0.15 c	163.38 ± 0.28 c	301.76 ± 1.42 c
BSB	2.06 ± 0.07 b	7.06 ± 0.12 b	20.23 ± 0.12 b	12.48 ± 0.13 b	4.18 ± 0.09 b	17.27 ± 0.08 b	225.82 ± 0.68 b	345.2 ± 2.68 b
BSA	2.73 ± 0.03 a	8.26 ± 0.24 a	24.36 ± 0.18 a	14.89 ± 0.06 a	4.59 ± 0.05 a	19.48 ± 0.12 a	249.17 ± 0.36 a	390.8 ± 3.87 a

Note: BS (*Botrytis cinerea* + sterile water), BSB (*B. cinerea* + strain *B. mojavensis* D50 before optimization) and BSA (*B. cinerea* + strain *B. mojavensis* D50 after optimization).

## Data Availability

Not applicable.

## Supplementary Materials

The following supporting information can be downloaded at: https://www.mdpi.com/article/10.3390/jof9050526/s1, Table S1: Primers used for quantitative real time PCR (qRT-PCR).
