# Supplementary material for: Adding Metal Ions to the Bacillus mojavensis D50 Promotes Biofilm Formation and Improves Ability of Biocontrol
Source: J Fungi (Basel). 2023 Apr 28;9(5):526. doi: 10.3390/jof9050526 (PMC10219225; doi:10.3390/jof9050526)
Supplement: Supplementary file 1 [file jof-09-00526-s001.zip › jof-2365159-supplementary.pdf]

Table S1 Primers used for quantitative real time PCR (qRT-PCR).

Gene	Forward (5′-3′)	Reverse (5′-3′)
luxS	AAAGTGGGAACAGACGGCGTTG	AGTGTGTGAATGGTGTCAGGCTTC
SinR	CTCCGCTGTTCTGGACGTCT	ACCCCGGATGTCATCGCATC
FlhA	TTCTTCTTTGCTCGCCTGCTGTC	CCACCACTCCAATGCTCTGTACTTC
tasA	GCACTCGGTTTAGCATTA	CTTCCGCAGATTCATTTC
16s rDNA	GGTGTAGCGGTGAAATGCGTAGAG	CATCGTTTACGGCGTGGACTACC
